# Supplementary figures and images for: Extracellular Matrix Deposition Defines the Duration of Cell Sheet Assembly from Human Adipose-Derived MSC
Source: Int J Mol Sci. 2023 Dec 1;24(23):17050. doi: 10.3390/ijms242317050 (PMC10707034; doi:10.3390/ijms242317050)

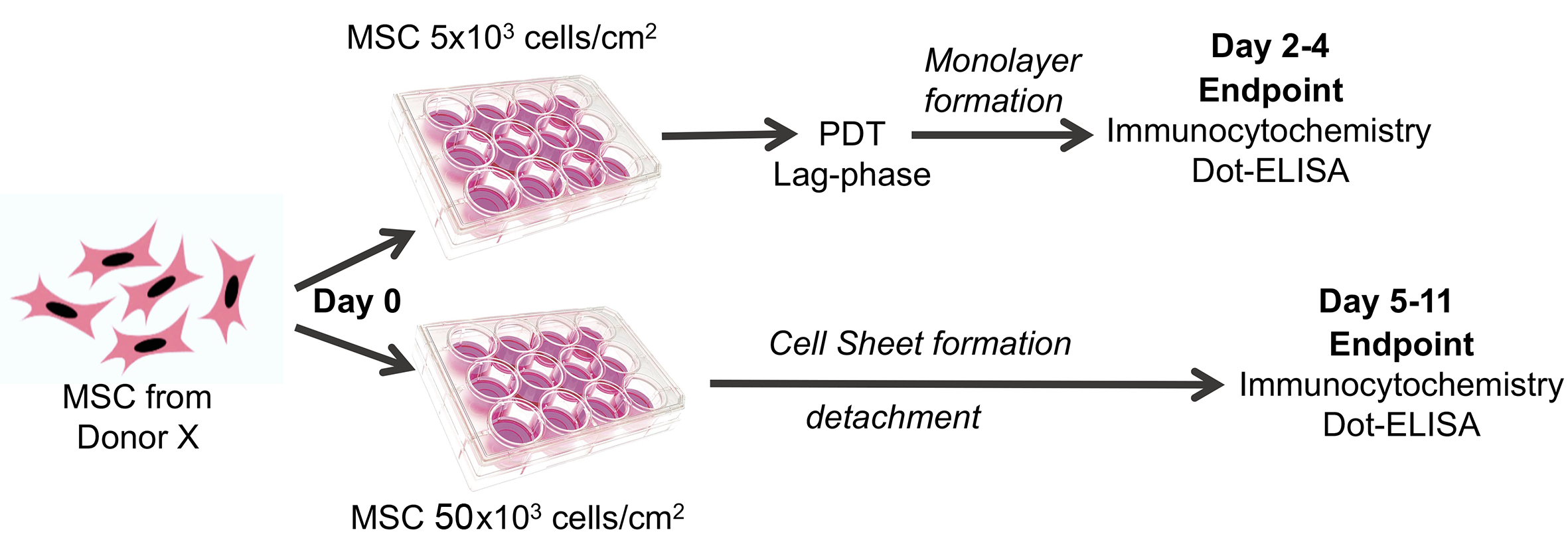

Supplement: Supplementary file 1 [file ijms-24-17050-s001.zip › ijms-2665213-supplementary.tif]
